# Is social media a promising global health center for the concussed? A scoping review of concussion coverage across social media

**DOI:** 10.34172/hpp.025.44332

**Published:** 2025-11-04

**Authors:** Aysha Jawed, Aryan Shabanpour, Nandita Gupta, Dennis Tudor, Yusuf Ghandi, Aria Mohebi

**Affiliations:** ^1^Department of Pediatrics, Johns Hopkins University School of Medicine, Baltimore, MD, USA; ^2^Department of English, University of Maryland, College Park, College Park, MD, USA

**Keywords:** Concussion, Social media, Mild traumatic brain injury, Prevention, Head injury, Clinical practice guidelines, Chronic traumatic encephalopathy

## Abstract

**Background:**

There are many risk factors that heighten severity and susceptibility of concussion which have come into heightened awareness attributed to more revelations across the concussions landscape in recent years in light of news media, cinema, landmark legal cases, and continued research findings.

**Methods:**

This review specifically focused on identifying concussion-related information accessed by different audiences on a range of social media platforms. The goals of this review were to synthesize the existing state of concussion coverage across social media platforms from all published studies to date as the basis to inform directions for patient and family education, clinical practice and future research on improving concussion care and treatment.

**Results:**

Findings revealed a wide range of consumer and professional sources publishing content on concussions. News stories and testimonials were the most widely accessed formats across two studies. YouTube, Twitter, Facebook, Instagram, TikTok, Pinterest, and Flickr were the social media platforms examined across these studies. Post-concussive symptoms and concussion treatment approaches were widely covered across two studies followed by prevention in three studies. Focus on situational and contextual factors of concussions (e.g. setting, surface and trauma-related considerations) were missing from findings in the studies across this review.

**Conclusion:**

Clinical and educational implications and recommendations for future ways to harness the potential of social media in improving concussion care and treatment are also presented. Increased content on concussion prevention could yield value in addressing modifiable risk factors for concussion as the basis to reduce the rates of this longstanding public health complexity.

## Introduction

 Brain injuries from trauma represent a longstanding and multifaceted public health complexity worldwide. Concussions form an integral part of this epidemic and represent one of the most prevalent and preventive types of brain injuries.^1^ Across the world, there are nearly 56 million mild traumatic brain injuries (oftentimes in the form of concussions) sustained annually.^2^ Among them, several could also represent repeated concussions. With growing research and ongoing revelations in findings on concussions, there is now more known about the long-term post-concussive effects from repeated head trauma including chronic traumatic encephalopathy (CTE), other neurogenerative diseases, death, increased rate of disability, and impact on functioning, activities of daily living and memory.

 As news stories and reports have sensationalized coverage of the aftereffects of repeated concussions over the past decade especially across the professional athletics landscape, examining coverage of this content from the extension of traditional media onto social media is timely. In this contemporary digital era, an uptake in typologies of social media platforms have offered consumers and professionals a wider range of options in accessing trending news and connecting with their networks and influencers around the globe.^3^ Furthermore, an increase in digital communication for dissemination of health-related content also is attributed to the growing number of social media platforms, thereby expanding both prevalence and utilization of social media for knowledge uptake in the healthcare landscape across a wider range of patient and provider populations worldwide.^4^ Of note, concussions have formed a trending headline in the news intermittently over the past decade, likely since the release of the movie Concussion starring Will Smith, revelation of new research findings on CTE, increased testimonials from athletes on CTE, resulting litigation, and in turn revisions to concussion protocols and clinical practice guidelines. Taking all of this into consideration, examining concussions as a trending health related topic is timely.

 Concussions represent the longstanding silent brain injury epidemic that is multifactorial and pervasive across the lifespan. Finding creative ways to mitigate it further remains a public health priority. Tapping into some of the contemporary digital tools in this wireless world could be a viable direction to explore ways to address the epidemic. In this digital era, there are many different social media platforms that include YouTube, Facebook, Twitter, Instagram, Flickr, Pinterest, Snapchat, TikTok among more. Based on a recent research study conducted by the Pew Research Center, YouTube, Facebook, and Instagram are the most widely popular ones at this time.^5^

 As a path to determine how informatics through data captured via social media could inform directions in concussion care, management, prevention, and education, it is crucial to first examine the existing state of coverage on concussions across this communication medium. Overall, social media has heightened awareness on risk factors, strategies as well as population-based considerations for concussions.^6-14^ Online support networks on social media have also contributed towards heightening knowledge uptake on concussions through facilitating exchange of strategies, experience and mutual support among patients and their families. One study examined exchange of this content across 17 Facebook groups.^9^ However, credibility of content on CTE as a causal or contributing factor for concussions, largely from testimonials of former professional athletes on social media (e.g. videos on YouTube) remains unclear as mechanisms of concussions are multifactorial. This content may not always provide a balanced overview of risk, causal, and contributing factors for concussions. It follows that this content yields implications for propagation of misinformation and disinformation. Prior reviews have examined published studies on trending health complexities (e.g. autism and antivaccine messages).^15,16^ Notably to date, no review has consolidated findings on sources, formats and content on concussions covered across social media sources. It follows that the present study will seek to fill this gap in the brain injury research landscape to ultimately determine a pathway to harness the potential of social media in disseminating best practices, reliable treatment approaches and considerations on increased severity and susceptibility of concussions (risk factors). Findings from this review will also yield insights on tapping into informatics as a resource in concussion care. In turn, the goals of this review are the following: 1) synthesize the existing state of knowledge, research and practice of concussion coverage on social media across published studies; 2) present the sources, format and content covered in these studies on concussions; 3) identify the current gap based on the depth and breadth of content covered and uncovered; and 4) propose recommendations for future research and practice to inform clinical care, research and education for concussion management and prevention.

## Methods

###  Search strategy

 A comprehensive search of the academic literature on concussion coverage across multiple social media platforms (YouTube, Twitter, Facebook, Instagram, TikTok, Pinterest, and Flickr) were conducted in January 2025. The medical, public health, educational, and psychosocial databases reviewed were EBSCO, Medline (Northfield, IL, USA), APA PsychInfo, Academic Search Premier, CINAHL, ERIC, and Cochrane Review. Singular and plural forms of concussion along with the names of each social media platform (Facebook, TikTok, Instagram, YouTube, Twitter, X, Reddit, Pinterest, Flickr) formed the key terms used across all of the database searches.

###  Eligibility criteria

 In this review, solely published studies on concussion coverage across seven social media platforms were examined. Given the focus of this review on academic literature across published studies, secondary and tertiary sources were excluded for analysis. Any sources that did not disseminate content on concussions were ultimately excluded.

###  Procedure

 Each published study in this review constituted the unit of analysis. These published studies comprised primary sources from descriptive, observational, cross-sectional and retrospective studies conducted on assessing sources, format, and content on concussions across the top widely trending social media platforms. The published studies in this review covered content pertaining to post-concussive symptoms, care, treatment, management and prevention considerations for concussions, as well as different situational and contextual factors surrounding concussions. The PRISMA checklist for scoping reviews thoroughly guided the assessment of quality and identification of any systematic biases for each of the studies integrated into this review.

 Six authors independently reviewed all of the titles and abstracts emerging across each database. Discrepancies concerning utility and inclusion of full vs. partial text sources as well as coverage of concussion-related content were resolved through active discussions among the team. Each author subsequently abstracted data independently across all studies in this review, specifically on sources, formats, and content on concussions presented in each source. Results were compared among the authors and any differences were fully resolved through consensus.

## Results

 Across the databases reviewed, a cumulative total of 91 records were identified from the past 50 years. 24 of these records were duplicates and subsequently excluded. Among the remaining 67 records, 19 of them were excluded for either missing full-text, referenced different head injury topics, and goals of research diverged from assessing concussion considerations. In turn, 48 full-text articles were thoroughly examined for inclusion in this scoping review.

 Among them, 39 were further excluded for one or more of the following reasons: (1) variation in study design; (2) secondary sources solely reporting information; (3) divergence in research goals; and (4) different topics covered that deviated from concussion-centered content.

 Nine articles ultimately met the criteria for concussion coverage across YouTube, Twitter, Facebook, Instagram, TikTok, Pinterest, and Flickr as presented in Table 1.^6-14^ Each study involved conducting cross-sectional and descriptive content analyses. A wide range of content was also covered across studies including concussion care, treatment, management and prevention considerations, post-concussive symptoms, and situational and context factors (e.g. setting, surface and trauma). All of the content examined across the sources in this review were in English. A comprehensive breakdown of the sources and formats of concussion-related content covered across the widely trending social media platforms from the published literature can be found in Table 1. Concussion content covered across these social media platforms in the reviewed studies are delineated in Table 2.

**Table 1:** Sources and format of concussion content across published social media studies

**Content **	**YouTube **	**Twitter**	**Facebook**	**Instagram**	**TikTok**	**Pinterest**	**Flickr**
Sources	News media^6^; consumers^6,7^; government^6^; advocacy groups^6^; television^7^; other internet users^7^	Doctors^13^; researchers^13^; health organizations^13^; consumers^13^		News media^8^		News media^8^	News media^8^
Format	News stories^6^; advertisements^6^	Testimonials^12^; news stories^12^; tweets^12,13^	Testimonials^9^	Images^8^; infographics^8^		Images^8^; infographics^8^	Images^8^; infographics^8^

**Table 2:** Concussion content covered across published social media studies

**Content category **	**YouTube **	**Twitter**	**Facebook**	**Instagram**	**TikTok**	**Pinterest**	**Flickr**
Post-concussive symptoms	Loss of consciousness^10,11^; Fencing Response^10,11^; seizures^11^; snoring^11^; crying^11^; vomiting^11^; residual and long-term effects of concussions^7^; physical aftereffects^7^; cognitive and emotional symptoms^7^; memory^7^; headaches^7^; dizziness^7^; clonus^10^; tonic motor symptoms^10^; eye deviation^10^; convulsions^10^; head wound^10^; disorientation^10^						
Concussion care, management and treatment	Seeking immediate and after care^7^; academic, athletic and work accommodations^7^; treatment approaches^6^; signs and symptoms^6,7^	Information on needing to stay awake during the acute phase of recovery following concussion^12^; management approaches^12^; awareness of concussions driven by the National Football League^12^; hospitalization^12^; cessation from sports^12^		Guidelines and policies^8^; concussion education^8^	Concussion facts^14^	Guidelines and policies^8^; concussion education^8^	Guidelines and policies^8^; concussion education^8^
Prevention	Helmet7,10; preventive measures^6^						
Populations	Children^11^; teenagers^11^; adults^11^						
Trauma	Assaults^10,11^; automobile accidents^11^; struck by object^11^						
Surface of Concussions	Collisions^10^; contact with concrete surface^10^; contact on wood, ice, ground and mixed martial arts mat^10^						
Athletics	Recreational/sports-related injuries^6^; concussions sustained from athletics^11^; cited sports as the cause of concussions^7^; concussive convulsions from athletics^10^						

###  Sources

 Sources were covered across several published studies reviewing concussion-related content on YouTube. News media sources posted videos in one study.^6^ Consumers created videos in two studies.^6,7^ The government also published videos in one study.^6^ Advocacy groups also developed videos in this study.^6^ Television sources posted videos in one study.^7^ Other internet users published videos in the same study.^7^ In another study that examined tweets posted on Twitter, doctors, researchers, health organizations and consumers authored these tweets.^13^ News media sources created content across Instagram, Pinterest and Flickr in another study.^8^

###  Formats

 There was also a diversity of formats reviewed across published studies examining concussion-related content on social media platforms. News stories were featured in two studies.^6,12^ Advertisements were presented in the same study.^6^ Testimonials were portrayed in two studies. ^9,12^ Tweets were depicted in two different studies.^12,13^ Images were delineated in one study examining content pertaining to concussions across Instagram, Pinterest and Flickr.^8^ Infographics were also covered in this study.^8^

###  Content

 Several studies that examined content related to concussions on YouTube uncovered a wide range of post-concussive symptoms. Loss of consciousness was covered in two studies.^10,11^ Fencing response was depicted in the same two studies.^10,11^ Vomiting was portrayed in one study. ^11^ Seizures were also examined in this study.^11^ Snoring was also reviewed in the same study.^11^ This study also presented content on crying.^11^ Residual and long-term effects of concussions were presented in one study.^7^ Physical aftereffects were delineated in the same study.^7^ Cognitive and emotional symptoms were also featured in this study.^7^ Memory was also represented in the same study.^7^ Headaches were also reviewed in the same study.^7^ Dizziness was also covered in this study.^7^ Clonus was examined in one study.^10^ Tonic motor symptoms were also depicted in the same study.^10^ Eye deviation was also portrayed in this study.^10^ Convulsions were also reviewed in the same study.^10^ Head wounds were also represented in this study.^10^ Lastly, disorientation was examined in the same study.^10^

 Concussion care, management and treatment were also covered across several studies examining content among different social media platforms. Seeking immediate and after care was explored in one study.^7^ Academic, athletic and work accommodations were presented in the same study.^7^ Treatment approaches were reviewed in another study.^6^ Signs and symptoms were also examined in this study.^6^ Another study yielded content on needing to stay awake during the acute phase of recovery following concussion,^12^ suggesting misinformation. Management approaches were also covered in this study.^12^ Also, awareness of concussions spearheaded by the National Football League was also depicted in the same study.^12^ This study also covered hospitalization and cessation from sports as concussion management strategies to improve recovery.^12^ In one study, guidelines and policies related to concussion care, treatment and management were reviewed across Instagram, Pinterest, and Flickr.^8^ This study also presented content on concussion education across these social media platforms.^8^ Concussion facts were also covered in one study examining content on TikTok.^14^

 Prevention was covered in published studies that examined concussion-related content on YouTube. Helmets were featured in two studies.^7,10^ Preventive measures without specification were covered in another study.^6^ One study on YouTube examined concussions among child, adolescent and adult populations.^11^

 Trauma was depicted across published studies on YouTube. Assaults were covered in two studies.^10,11^ Automobile accidents were also portrayed in the same study.^11^ Stuck by object as the cause of concussion was also presented in this study.^11^

 Surface of concussions was reviewed in one published study examining content pertaining to concussions on YouTube. Collisions were featured in one study.^10^ Contact with concrete surface was depicted in the same study.^10^ Lastly, contact on wood, ice, ground and mixed martial arts were also presented in this study.^10^

 Athletics were also covered across four published studies reviewing concussion coverage on YouTube. Recreational and sports-related injuries were explored in one study.^6^ Concussions sustained from athletics were portrayed in another study.^11^ Sports were cited as the cause of concussions in a different study.^7^ Concussive convulsions attributed to athletics were depicted in a study.^10^

## Discussion

 Overall, nine published studies examined sources, format and content on concussions across trending social media platforms. Among these studies, YouTube was the most frequently assessed, spanning four studies that encompassed widely viewed videos on concussions as well as specialized considerations. A wide range of post-concussive symptoms were also covered across the published studies reviewing content pertaining to concussions on YouTube. There were several considerations related to concussion care, management, and treatment reviewed across the majority of the studies. Notably, concussion prevention was also solely featured across published studies examining content on YouTube. There was scant content on misinformation pertaining to signs, symptoms, and recovery for concussions present across all of the studies. Gender differences in concussion prevalence were also not reviewed among most of the studies. News media sources generated engagement and encapsulated a wide variety of formats including news stories, testimonials, interviews, infographics, and images. Different consumer and professional sources published content across social media platforms on a variety of concussion considerations. There was limited coverage of settings, surfaces, and trauma related situational and contextual factors surrounding concussions across the widely viewed and specialized content examined across these published studies.

 Notably, news media comprised the primary source of content represented across the published studies in this review. News media has the potential to draw in more consumers given there are opportunities to simultaneously present stories, testimonials, still images, infographics, interviews, and other formats in covering segments not only for concussions but a range of different trending content. Of note, news media is not considered at this time to be an evidence-based source of disseminating education and recommendations for concussion care and treatment. However given its increased prevalence across studies, it could be helpful to further explore directions in partnering with news media sources in future concussion awareness campaigns, uptake of knowledge on revisions in future iterations of clinical practice guidelines and protocols for concussion care and management, and in revelations from research on ongoing mechanisms and aftereffects of concussions for diverse patient populations.

 In addition, there was not much representation of health promotion content in the form of concussion-centered campaigns on social media across the published studies reviewed (for e.g. the Heads Up Campaign from the Centers for Disease Control and Prevention, global concussion awareness campaign jointly spearheaded by the Fédération Internationale de Football Association and World Health Organization). Campaigns represent a health promotion intervention that yields potential to cast a wider net and can be extended from traditional sources of media (e.g. mass media, outdoor advertisements, printed materials) onto nontraditional sources of media (e.g. social media). It follows that integrating campaign-centered content for concussions on social media could be another direction to heighten knowledge and awareness on concussion prevention and treatment among consumers and healthcare professionals worldwide.

 It is also crucial to note that the majority of the published studies examined content across YouTube, whether related to the widely viewed content on concussions or specific post-concussive symptoms. Limited studies were published on TikTok, Flickr, Instagram, Facebook, and Pinterest. There were a few studies covering concussion related content on Twitter. These findings suggest that knowledge dissemination on concussions across social media is still in nascent stages given that this content does not encompass the growing social media landscape that accounts for a wider range of digital communication platforms. Furthermore, content posted on one social media platform oftentimes can be found on another platform. It follows that capturing content that is more widespread across platforms could help determine credibility and reliability of this pervasive content. In addition, this information could further help determine whether the content is in line with existing clinical practice guidelines and concussion protocols in care and treatment considerations, thereby seeking to identify possible risk for misinformation and disinformation to more consumers. Further assessment of content to determine its reliability from alignment with guidelines will help identify targets for demystifying propagation of misinformation and disinformation on concussion care considerations across social media.

 Of note, only published studies reviewing content on YouTube covered a variety of post-concussive symptoms. Across the rest of the studies, content on aftereffects was vague and nonspecific. It follows that increased research on exploring and assessing content across the rest of the social media platforms to determine veracity of content disseminated over time could yield directions in improving concussion education and management across digital communication mediums more effectively for our global population worldwide. Facebook, Instagram, and Twitter are also rising and trending social media platforms.^5^ In turn, tapping into the depths of their content through conducting successive studies could help reveal specific considerations in concussion care for patients worldwide.

 Surface, setting, trauma, and athletics were also only focused on as risk factors for concussions in the published studies examining content on YouTube. Each of these risk factors heightens not only susceptibility but severity of injury and are the basis for several of the recommendations across clinical practice guidelines and concussion protocols (American Association of Neurological Surgeons, CDC, American Academy of Pediatrics, Academy of Neurologic Physical Therapy).^17-19^ However, given their limited coverage across the studies in this review, it is crucial to build on this research to further assess their visibility across the social media landscape. News media and television seek to sensationalize trauma which in the concussion context can encompass a range of different surfaces (e.g. concrete, roadway, rocks, grass, hardwood) and settings (e.g. field, gymnasium, school, etc) as well as other situational and contextual factors (e.g. struck by individual or object, fall, collision, motor vehicle or dirtbike accident, etc). These stories drew in more viewers across the published studies on YouTube and Twitter. It follows that finding ways to either draw on the strengths of integrating content disseminated by these sources or partnering with these sources could heighten awareness and support of necessitating timely and needed prevention and intervention of concussions for our global population.

 Notably given publication dates of these studies or their specific focus on specialized considerations pertaining to concussions, none of them captured the wider series of case studies and happenings that have unfolded in the aftermath of the movie Concussions featuring Will Smith, the increased revelations by former and current athletes on suffering from CTE attributed to repeated concussions, litigation faced by major league athletics organizations stemming from the sequelae of concussions among professional athletes, and subsequent revisions in clinical practice guidelines and concussion protocols. It follows that a more up-to-date exploration of sources, format and content on concussion considerations across any of the social media platforms reviewed in this study could yield information on changes in the concussion care landscape on social media as a rising health communication medium.

 It is crucial to also note the limitations in this review. First, in light of the scoping, descriptive, and narrative design of this review, rigorous composite statistical analyses were not conducted and in turn, there was no critical examination of study biases that are part of a systematic review. In addition, all sources examined in this review were in English, thereby precluding any pertinent sources published in different languages. Also, solely published studies were reviewed which could also preclude any secondary sources involving a review or compilation of findings on concussions covered on social media. It is also imperative to acknowledge that there could be potential bias in sample size and selection methods and inconsistencies in the search strategies utilized to access content across studies. It follows that widely viewed and accessed content may not have been fully captured in one or more of the samples of videos, tweets, open-ended posts, and hashtags given that the search algorithm across these studies remains unknown. In addition, each of the published studies involved cross-sectional study designs which offered a snapshot of content at one conceptual point in time. Given the ever-evolving landscape of social media and latest updates in trending content, coverage of concussion-related content at this current point in time could vary substantially compared to prior widely viewed posts across different social media platforms. In addition, the number of views, tweets, likes, and comments that these posts generate are always changing. It follows that successive sampling of posts through ongoing content analyses and descriptive, exploratory research could further assess any changes in concussion coverage across all of the social media platforms reviewed in this study. Lastly, no published studies have examined concussion-related content to date on Linkedin, WhatsApp, Redditt, and any other social media platforms outside of the ones examined across this review. Future studies could conduct content analyses on concussion related content across these social media platforms to ultimately yield a more comprehensive understanding of the widely covered content and its alignment to clinical practice guidelines in concussion prevention and treatment.

## Conclusion

 Concussions continue to remain a longstanding silent brain injury epidemic worldwide. Social media yields promise in disseminating content on concussion education, treatment and management globally given increased access to technology. The data is not yet compelling to suggest that digital communication is an evidence-based intervention for disseminating concussion-related knowledge and contributing to clinical practice. Continuing successive research on examining content pertaining to concussions across the diversity of social media platforms could yield a viable point of comparison with existing clinical practice guidelines and concussion protocols. Content published by professional and governmental sources that also extend concussion awareness campaigns onto social media can help increase knowledge uptake of sound recommendations worldwide for concussion care, prevention and treatment. It follows that future updated content on concussions across social media could be further evaluated for credibility and reliability, specifically in regard to alignment with these clinical recommendations as the basis to not only assess risks for propagation of misinformation and disinformation but also to make the case that digital communication could further be assessed as an evidence-based practice in concussion treatment and prevention for our global population.

## Competing Interests

 The authors have no competing interests to declare.

## Ethical Approval

 This article does not contain any studies with human participants or animals performed by any of the authors.
